# Prebiotic Effects of Seaweed Polysaccharides in Pigs

**DOI:** 10.3390/ani11061573

**Published:** 2021-05-27

**Authors:** Carlo Corino, Alessia Di Giancamillo, Silvia Clotilde Modina, Raffaella Rossi

**Affiliations:** Dipartimento di Medicina Veterinaria, Università degli Studi di Milano, Via dell’Università 6, 26900 Lodi, Italy; carlo.corino@unimi.it (C.C.); alessia.digiancamillo@unimi.it (A.D.G.); silvia.modina@unimi.it (S.C.M.)

**Keywords:** pig, polysaccharides, prebiotics, seaweed

## Abstract

**Simple Summary:**

In recent decades, the ban of antibiotic growth promoters together with the reduction in the feed of trace minerals with antimicrobial properties such as zinc and copper, has increased the demand to identify organic alternatives with antimicrobial properties that may improve the production efficiency and sustainability in an antibiotic-free system. The sustainability of pig production can be enhanced, by increasing the feed efficiency, modulating the microbiota, and reducing the impact of different diseases through the use of natural substances. Considerable research has focused on the gut environment and microbiota modulation as key to boosting pig health. Sustainable dietary interventions that positively modulate the gut environment and microbiota in pigs are required to enhance pig health and welfare. In the field of natural substances, seaweed and their bioactive compounds have assumed particular importance as feed ingredients for pigs. In fact, seaweeds include numerous bioactive substances with prebiotic, anti-microbial, antioxidant, anti-inflammatory, and immunomodulatory activities. The present paper reviews the prebiotic effects of seaweed polysaccharides in pigs.

**Abstract:**

To ensure environmental sustainability, according to the European Green Deal and to boost the One Health concept, it is essential to improve animals’ health and adopt sustainable and natural feed ingredients. Over the past decade, prebiotics have been used as an alternative approach in order to reduce the use of antimicrobials, by positively affecting the gut microbiota and decreasing the onset of several enteric diseases in pig. However, dietary supplementation with seaweed polysaccharides as prebiotics has gained attention in recent years. Seaweeds or marine macroalgae contain several polysaccharides: laminarin, fucoidan, and alginates are found in brown seaweeds, carrageenan in red seaweeds, and ulvan in green seaweeds. The present review focuses on studies evaluating dietary seaweed polysaccharide supplementation in pig used as prebiotics to positively modulate gut health and microbiota composition.

## 1. Introduction

Gut health, which is described as a generalized condition of homeostasis in the gastrointestinal tract [1], has been recognized as playing a key role in maintaining pig health. In fact, the gut plays an important role in efficient feed digestion and absorption, for the protection of the gut barrier, the microbiota composition, and the improvement in the immune status [2]. In fact, commensal bacteria such as *Lactobacilli* and *Bifidobacteria* are necessary to sustain the host immune system, protecting against the colonization of opportunistic pathogens [3].

Since the ban on in-feed antibiotics, reliable dietary interventions are needed that are capable of sustaining pig performance and improving gut health, by minimizing the use of antimicrobials. A large amount of evidence has reported the beneficial effects of some feed ingredients or additives in modulating gut health and microbiota in pig. 

The review by Xiong et al. [2] focused on the effects of several feed ingredients or additives such as functional amino acids, natural extracts, and short-chain fatty acids and prebiotics on gut health in weaned pigs. 

Over the past few decades, prebiotics have been used as an alternative approach aimed at reducing the use of antimicrobials, by positively affecting the gut microbiota and decreasing the onset of several enteric diseases in pig [4]. However, dietary supplementation with seaweed polysaccharides as prebiotics, has also gained attention in recent years. In fact, natural bioactive compounds have been considered as attractive dietary interventions in pig in order to ensure environmental sustainability, in line with the European Green Deal plan and to improve animal health according to the One Health approach.

Marine macroalgae, or seaweeds, are classified as brown algae (*Phaeophyceae*), red algae (*Rhodophyta*), and green algae (*Chlorophyta*) and include thousands of species. The chemical composition and the bioactive metabolite content of several species have been extensively studied, along with the variations related to species and genera, harvesting season, environmental conditions, and geographical location [5,6]. Seaweeds also contain large amounts of carboxylated and sulfated polysaccharides, with important functions for the macroalgal cells including structural and energy storage [7]. Seaweed polysaccharides are safe, environmental-friendly, and economical natural polymers. Seaweed polysaccharides, such as fucoidan, laminarin, ulvan, carrageenan, and alginates, show several biological activities in vitro and in vivo studies [8,9]. In fact, polysaccharides and oligosaccharides originating from seaweeds have been shown to regulate intestinal metabolism and fermentation and reduce the adhesion of pathogenic bacteria [10]. Several seaweed polysaccharides have also shown anti-inflammatory, antiviral, and antioxidant activities [11]. Considering the above mentioned properties, the present paper reviews the prebiotic effects of seaweed polysaccharides in pig nutrition.

## 2. Seaweed Polysaccharides

The polysaccharides contained in brown, red, and green seaweeds present different bioactive molecules such as fucoidan, laminarin, alginate, ulvan, and carrageenan, which are reported in Table 1.

The yield of seaweed polysaccharides varies in relation to the species-growing conditions, extraction method, such as solvent concentration and extraction time [14]. The polysaccharide content of brown, red, and green seaweeds is reported in Table 2. The total polysaccharide content in seaweeds is highly variable, fluctuating from 4 to 80% of dry matter (DM), according to the data of Lafarga et al. [12].

In green seaweeds, the content ranges from 15 to 65% of DM with the highest value for *Ulva* spp., in red seaweeds from 53 to 66% of DM with the highest value in *Chondrus crispus*, and in brown seaweeds from 10 to 66% DM with the highest amount in *Ascophyllum nodosum* and *Saccharina* spp. [15]. 

Carrageenans and agars are the two main polysaccharides in red seaweeds, but porphyran and xylan have also been observed [16]. Carrageenans are sulfated polysaccharides, composed of d-galactose units, with a structural role, similar to cellulose in plants, and are present in some red algae, such as *Chondrus*, *Gigartina*, and *Hypnea* [17], with the highest amount in *Chondrus* and *Kappaphycus* spp. [18]. Agar is largely observed in the *Gelidium* and *Gracilaria* spp. and is composed of agarose and agaropectin [19]. Fucoidans, alginates, and laminarin are the main polysaccharides in brown seaweeds. 

Alginates are the main cell wall polysaccharides in brown algae, such as *Laminaria* spp., *Fucus* spp., *Ascophyllum nodosum*, and *Macrocystis pyrifera* [20]. Besides alginates, fucoidans are cell wall water-soluble polysaccharides in brown seaweeds, containing L-fucose and sulfate groups, in addition to monosaccharides such as mannose, glucose, xylose, and glucuronic acid [21].

**Table 2:** Polysaccharides composition of brown, red, and green seaweed (g kg^−1^ DM) ^1^.

Seaweed	Polysaccharides, %	Alginates	Carragenan	Fucoidan	Laminarin	Ulvan	References
**Brown**							
*Ascophillum nodosus*	62 (42–70)	285 (240–330)	-	75 (11–120)	118 (12–120)	-	[8,18,22,23,24,25]
*Laminaria hyperborea*	39.9 (14.4–65.5)	215 (22–408)	-	30 (20–40)	125 (0–320)	-	[8,18,26,27,28]
*Laminaria digitata*	57.3 (44–70.7)	435 (350–520)	-	49.5 (22–112)	120 (0–350)	-	[8,18,26,27,29,30,31]
*Laminaria* sp. *	45 (13–77)	309 (225–343)	-	147.5 (22–550)	153 (62.4–340)	-	[8,26,29,32,33,34,35]
*Fucus* sp. ^#^	57 (34.5–66)	162	-	105 (11–200)	2.3 (0.4–3.8)	-	[18,23,24,27,34,36,37,38]
*Sargassum* sp. ^⁑^	36 (4–68)	296 (93–499)	-	38 (31–45)	3 (0–6)	-	[18,34,36]
*Saccharina* sp. **	69 (58–80)	242.5 (200–285)	-	33 (13–80)	97.5 (0–330)	-	[5,8,18,23,27,29,39]
*Undaria pinnafitica*	40 (35–45)	425 (340–510)	-	219 (30–690)	30	-	[8,18,29,40]
**Red**		-	-	-	-	-	
*Chondrus crispus*	60.5 (55–66)	-	439.5 (338–510)	-	-	-	[18,34,41]
*Kappaphycus alvarezii*	58 (53.5–64)	-	448.5 (187–756)	-	-	-	[32,41,42,43]
**Green**						-	
*Ulva* sp. ^§^	42 (15–65)	-	-	-	-	176 (11–400)	[18,34,44,45,46,47,48]

^1^ Data are reported as mean values and range (minimum-maximum). * Values from *Laminaria claustonii* and *japonica*. ^#^ values from *Fucus vesciculosus*, *serratus*, *spiralys*. ^⁑^ Values from *Sargassum patens*, *hemifhyllum*, *henslowianum*. ** Values from *Saccharina longicruris*, *latissima*, *cichorioides*, *japonica*, *longissimi*. ^§^ Values from *Ulva armoricana*, *lactuca*, *intestinalis*, *meridionalis*, *pertusa*. - Polysaccharides not present in the considered seaweed.

Laminarin, also called laminaran, is a storage polysaccharide in brown seaweeds which is composed of (1–3)-β-d-glucan. The laminarin structure differs in the degree of branching and polymerization. The highest laminarin content is found in *Laminaria* spp. and *Saccharina* spp. (32% DM), however it is also present in small amounts in *Ascophyllum*, *Fucus*, and *Undaria* spp. [18]. Ulvan is the constituent of the cell wall of green seaweeds and is constituted by β-(1–4)-xyloglucan, glucuronan, and cellulose in a linear arrangement [49]. The ulvan content varies from 2.7% DM in *Ulva flexuosa* to 40% DM in *Ulva Armoricana* [48].

## 3. Seaweed Polysaccharides as Prebiotic

Carbohydrates, which are indigestible to hydrolytic enzymes and are fermentable, are considered as prebiotics. They must not be digested or adsorbed in the first tract of the gut, however they should be fermented in the colon by *Lactobacillus* and *Bifidobacterium*, enhancing their growth and decreasing the concentration of other invading pathogens in the large intestine [50]. Digestion can affect the seaweed polysaccharide activity as prebiotics. The first step is to verify the resistance to hydrolysis by acids and enzymes in in vitro conditions.

Laminarins from different seaweeds vary in terms of the structural characteristics such as the degree of polymerization and the presence of inter-chain hydrogen bonds. These complex structures are resistant to hydrolysis in the first tract of the gut and are studied as dietary fibers [51]. In brown seaweed, polysaccharide laminarins were indigestible in an in vitro model with hydrochloric acid and enzymes [52]. In addition, laminarin from Laminaria saccharina and digitata were fermented, producing short-chain fatty acids (SCFA) [53]. Another study reported that SCFA that are produced from the fermentation of *Laminaria digitata* and *Undaria pinnatifida* are not metabolized well compared to the sugar beet fibers [54]. 

In a simulated digestion model, *Ascophyllum nodusum* was fermentable, thus producing SCFA and reducing the concentration of total anaerobe bacteria such as *Escherichia coli* and *Streptococcus* in batch systems inoculated with porcine cecal suspensions [55]. The same result was obtained in the *Porphyra* spp., a red seaweed with galactoside, which was not digested by salivary, gastric, pancreatic, or intestinal enzymes and was fermentable by *Bifidobacterium* [56]. 

Alginate, agarose, and carrageenan, from brown and red seaweeds are not digested but fermented by the gut bacteria [57]. Oligosaccharides enzymatically hydrolyzed from alginate, agarose, and carrageenan have shown prebiotic activities, stimulating *Bifidobacteria* and *Lactobacilli* growth and producing SCFA [58,59]. A recent in vitro study evaluated the fermentability of the three aforementioned oligosaccharides using pig fecal microbiota. The data reported that all the oligosaccharides considered were able to enhance SCFA concentration, in particular butyric acid. A positive shift in gut microbiota was also observed for alginate and agarose oligosaccharides with a decrease in pathogenic bacteria [60].

Ulvan, the main sulphated polysaccharide in green seaweeds, is a water-soluble dietary fiber resistant to digestive tract enzymes, however it is poorly fermented by colonic bacteria and thus in its form, it is not studied as a prebiotic, but could be hydrolyzed to bioactive oligosaccharides [61]. Low molecular weight *Ulva armoricana* aqueous extract, whose main component is ulvan, has shown antibacterial activity against several Gram-positive and Gram-negative bacterial strains [62].

Several in vivo studies in rats, highlighted the prebiotic activity of seaweed polysaccharides, as reviewed by O’Sullivan et al. [63]. In fact, it is reported that in rats, dietary supplementation with alginate oligosaccharides or agarose hydrolysate increased the cecum *Bifidobacterium* and *Lactobacillus* count. Another study on rats reported that dietary supplementation with *Chondrus crispus* increased the SCFA concentration and reduced the *Clostridium* and *Streptococcus* concentration [64]. In mice fed *Laminaria japonica*, a higher production of SCFA and butyric acid was observed, together with a decrease in Clostridium, Escherichia coli, and Enterobacter [65]. Considering the data on in vitro studies and laboratory animals, several studies on dietary supplementation with seaweed polysaccharides have been conducted in pigs to improve microbiota composition, and reduce the onset of several diseases and the subsequent antibiotic treatment. 

## 4. Seaweed Polysaccharides as Prebiotics in Sows

The effects of algae polysaccharides as prebiotics in sows have been evaluated by several authors as presented in Table 3. 

The effects of polysaccharides in the gut are usually assessed by evaluating the SCFA content and the intestinal microbiota composition and/or the presence of beneficial bacteria [72]. The effects of dietary supplementation with seaweeds in sows can modulate the productive performances and health of lactating piglets, making them more resistant to pathogens.

Dietary brown seaweeds were evaluated as a prebiotic in field conditions. In the study by Leonard et al. [66] an antimicrobial effect of seaweeds was observed. In fact, the establishment of neonatal gut microbiota was mediated by the fecal microflora of sows or by the colostrum and milk composition. This early gut colonization is fundamental for the development of healthy microbiota and for modulation of the immune system [73].

In piglets after 9 days of weaning, a decrease in *Escherichia Coli* was observed, however the same trend was also detected for *Bifidobacteria* and *Lactobacillus*. Moreover, a positive effect on the intestinal morphology was observed in the treated piglets. Similar result on the modulation of the microbiota was observed in a subsequent study from the same authors [67]. A reduction in fecal *Escherichia coli* in sows may positively affect piglet’s microbiota with a lower *Enterobacteriaceae* count which influences the onset of enteric pathologies [74]. In fact, although the *Enterobacteriaceae* count was higher in sows receiving dietary laminarin and fucoidan, the diarrhea score was lower in piglets from treated dams [68]. A positive impact on the ileum morphology of piglets from treated sows has also been observed, with an increase in villi height at weaning. In the study by Bouwhuis et al. [70] a lower expression was observed of heat-labile enterotoxin and enteroaggregative heat-stable enterotoxin, which are responsible for the intestinal colonization of enterotoxigenic *Escherichia coli* and represent the most frequent cause of diarrhea. It has been reported that laminarin, which is a source of β-glucans, lowered intestinal *Enterobacteriaceae* and reduced *Escherichia coli* numbers in feces. This could be ascribed to the potential agglutination activity, as earlier observed for yeast β-glucans which prevent bacterial adhesion and the colonization of the epithelial mucosa [75,76].

An antibacterial activity of fucoidan has also been reported [77]. An increase in *Lactobacilli* count was also observed which was also shown to be helpful for gut health with a positive modulation of the immune system [78,79].

In the considered studies the effects of laminarin and fucoidan from *Laminaria* spp. on *Lactobacilli* population were inconsistent.

## 5. Seaweed Polysaccharides as Prebiotics in Post Weaning Piglets

Weaning is a critical phase in pig production, often characterized by high antibiotic and microelement use. In fact, at weaning the gastrointestinal tract and immune system of piglets are not yet fully developed and the social, environmental, and physiological challenges, predispose the piglets to dysbiosis [80]. These challenges lead to a lower feed intake and growth rate and a high incidence of post-weaning diarrhea (PWD) due to the presence of enteric pathogenic bacteria [81].

In fact, at weaning, a lower *Lactobacilli* count has been observed, with a high growth of facultative anaerobes bacteria such as *Enterobacteriaceae*, *Proteobacteriaceae*, *Clostridiaceae*, and *Prevotellaceae* [80]. After weaning, structural and functional alterations of the small intestine have also been observed with negative effects on the absorptive capacity [82].

Feeding strategies in the post-weaning phase can reestablish the gut eubiosis that was lost at weaning, aimed at restoring the Lactobacillus count, promoting the growth of beneficial bacteria that boost the mucosal immune system and lowering the pathogenic bacteria proliferation [83].

The role of diet in the post weaning health status is widely recognized, in fact feed ingredients and additives can exert selective pressure on the gut microbiota. It has also been reported that dietary fermentable carbohydrates play a key role in positively affecting the intestinal microbiota of post-weaning piglets [84].

Several studies have evaluated the effects of seaweed polysaccharides as prebiotics in post weaning piglets, as reported in Table 4.

The dietary inclusion of *Ascophillum nodosum* in the piglets’ diet can reduce the *Escherichia Coli* content in the small intestine of weaned piglets [85]. The *Lactobacillus/Escherichia coli* ratio in the small intestine was shown to increase in the piglets receiving dietary seaweeds suggesting a helpful microbial modification. A reduction in the *Enterobacteriaceae* count was also observed. These are opportunistic pathogens such as *Salmonella Typhimurium* and enterotoxigenic *Escherichia coli* (ETEC) that induce PWD in piglets [85]. No effects of dietary *Ascophillum nodosum* on the small intestinal morphology were observed but an increase in total SCFA and butyric acid content were reported [85,86]. Similar data on gut health improvement have been observed with dietary supplementation with *Laminaria* spp.

Laminarin and fucoidan, as sources of seaweed polysaccharides with prebiotic effects, are able to decrease fecal *Escherichia coli* counts in the feces, thus improving post-weaning piglet health with a positive effect on growth performance and gain to feed ratio [88,90]. An improvement in *Lactobacillus* count has also been detected [88,89,91,92,93].

It has been also reported that laminarin, modifying the resident microbiota, may indirectly enhance mucin synthesis and secretion, as adherence of beneficial bacteria to mucosal epithelia up-regulates the mucin production. An enhancement of cytokine gene expression was also observed after a lipopolysaccharide (LPS) challenge [101]. 

Fucoidan also supports *Lactobacillus* growth with a positive effect on feed digestibility [87,101].

The increase in butyric acid reported in several studies, is usually related to carbohydrate fermentation which has a positive effect on gut health [87,91,92,97]. However, in the study by Sweety et al. [91], after a *Salmonella typhimurium* challenge, proliferation of Salmonella shedding was observed after dietary supplementation with laminarin and fucoidan. This is probably related to the decreased content of propionic acid and increased content of acetic acid which can modulate the gene activation in *Salmonella* pathogenicity Island 1 (SPI−1) [102]. 

A recent study showed that the alginic acid oligosaccharide from seaweed, which contains 96% α-L-guluronic acid and 4% β-D-mannuronic acid, has antibacterial and anti-biofilm activities. These oligosaccharides can modulate mucosal cytokine expression and antibody production and promote the growth of *Lactobacillus* [103]. As recently reported, dietary seaweed alginic acid oligosaccharides in weaned piglets can boost the intestinal barrier integrity, upregulating occludin mRNA expression in the caecal mucosa and claudin—1 mRNA expression in the caecal and colonic mucosa [98].

A recent study in piglets also showed that supplementing a seaweed product in drinking water (Algo-Bio^®^) decreased the *Enterobacteria* resistance rate to tetracycline [104]. In addition, in piglets treated with a seaweed supplement, the antibiotic susceptibility test showed a high sensitivity of *Escherichia coli* to imipenem, amikacin, and netilmicin with low chloramphenicol resistance. In fact, in agreement with the data of Berri et al. [105], the use of seaweed in pig nutrition to overcome antibiotic resistance is promising.

## 6. Seaweed Polysaccharides as Prebiotics in Growing-Finishing Pigs

Table 5 reports the effects of seaweed carbohydrates as prebiotics in growing-finishing pigs. The antimicrobial properties of seaweed polysaccharides in growing-finishing pigs could be effective in reducing pre-slaughter enteric pathogens. 

In fact, several studies have reported a lower enteric *Escherichia coli* count in growing-finishing pigs fed *Ascophillum nodosus* or *Laminaria* spp. [101,106,107]. Dietary seaweed polysaccharides reduced intestinal and fecal *Salmonella Typhimurium* and lowered the colonic gene expression of pro-inflammatory cytokines in growing-finishing pigs after an experimental *Salmonella Typhimurium* challenge [108,109]. 

The effects of *Laminaria digitata* extract in wet, spray-dried, and freeze-dried forms have also been evaluated [110,111]. The laminarin and fucoidan from *L. digitata* in wet form showed a prebiotic effect in pig, enhancing the *Bifidobacterium sp.* count in the ileum. In fact, several technological processes, such as spray drying techniques, have been shown to reduce the functionality of some bioactive compounds [112]. An increase in the colon and cecum SCFA was also detected following dietary supplementation with laminarin [101] and fucoidan [113]. 

In the growing finishing phase, the decline in enteric *Escherichia coli* and *Salmonella Typhimurium* count and the improvement in beneficial bacteria, such as *Bifidobacterium* and *Lactobacillus*, suggest that brown seaweed polysaccharides are a sustainable dietary strategy to improve gut health, by modulating microbiota and reducing pre-slaughter pathogens.

**Table 5:** Prebiotic effects of seaweed in growing finishing swine.

Seaweed Supplement	Dose	Animal	Prebiotic Effect	Effect vs. Control %	Reference
*Ascophyllum nodosum*	Dried intact ^x^ (3–6–9 g kg^−1^)	Pig, 48.7 kg LW	Ileum: *Coliform* *Lattobacillus* *Adherent Lactob.* *Biphydobacteria*	−19% NS NS NS	[106]
*L. digitata*	Wet (W) or spray dried (SD) seaweed	Pig 14.5 kg LW	Ileum: *Bifidobacterium* *Lactobacillus* *Enterobacteriaceae*	+18% wet NS NS	[110]
*L. digitata*	Wet (W) or spray dried (SD) seaweed LAM + FUC (0.50–0.42 g kg^−1^)	Pig, 14.5 kg LW	Ileum: *Bifidobacterium* *Lactobacilli* spp.	+45% SD +66% W +31% SD +47% W	[111]
*L. digitata*	Seaweed extract ^y^ LAM (0.3–0.6 g kg^−1^)	Pigs, 18 kg LW	Colon: *Enterobacteriaceae* Caecum: SCFA	−29% LAM 0.3 −22% LAM 0.6 +9% LAM 0.3 +12% LAM 0.6	[101]
*L. hyperborea*	Seaweed extract LAM (0.30 g g kg^−1^) FUC (0.36 g kg^−1^) LAM + FUC (0.3 + 0.36 g kg^−1^)	Boars, 65 kg LW	Proximal colon: *Enterobacterium* spp. *Lactobacilli* spp. SCFA Distal colon: *Enterobacterium* spp. *Lactobacilli* spp. SCFA	+14% LAM + FUC +16% FUC +16% FUC +30% LAM + FUC +43% FUC +36% FUC	[113]
*L. hyperborea*	Seaweed extract ^z^ (0.7–1.4–2.8–5.6 g kg^−1^) LAM = 0.08–0.16–0.32–0.64 g kg^−1^ FUC = 0.06–0.12–0.24–0.48 g kg^−1^	Boars, 76 kg LW	Caecum: *Bifidobacterium* spp. *Enterobacterium* spp. Colon: *Entorobacteria* *Bifidobacterium* spp. *Lactobacilli* spp.	−3, +5, +2, +3% +3,− 4, −7, −4% −3, −9, −7, −2% +3, +5, +2, −3% 0, −1, −2, −3%	[107]
*Laminaria* spp.	Seaweed extract LAM + FUC (0.18 + 0.34 g kg^−1^)	Female pigs, 30.9 kg LW Challenge *Salmonella Typhimurium* 11 d	Caecum: *S. Typhimurium* *Lactobacillus* spp. Colon *S. Typhimurium* *Lactobacillus* spp.	−16% +3% −24% +1%	[108]
*Laminaria* spp.	Seaweed extract in diet LAM + FUC (0.18 + 0.34 g kg^−1^)	Female pigs, 30.9 kg Challenge *Salmonella Typhimurium* at 11 d	*Salmonella Typhimurium* Caecum Colon	−16% −24%	[109]

LAM: laminarin; FUC: fucoidan; SCFA: short-chain fatty acids. ^x^ The *Ascophillum Nodosum* seaweed extract (Kerry Enhancer, Kerry Algae, Curraheen, Tralee Co., Kerry, Ireland). ^y^ Purified laminarin (990 g/kg) laminarin was sourced from Bioatlantis Limited, Tralee, County Kerry, Republic of Ireland. ^z^ The seaweed extract contained laminarin (112 g kg^−1^), fucoidan (89 g kg^−1^).

## 7. Conclusions

Overall, the present data demonstrate that the prebiotic activity of seaweed polysaccharides could be used to improve pig health in several production phases, thus reducing the use of antimicrobials. The gut health enhancement and reduction in pathogenic bacteria in lactating and post weaning piglets are fundamental in modulating productive performance and health, by reducing gastrointestinal diseases and enhancing feed digestibility. In the growing-fattening phase, a modulation of microbiota and a reduction in pre-slaughter enteric pathogens have also been observed.

In conclusion, dietary supplementation with brown seaweed polysaccharides seems to be a valid strategy to modulate microbiota, making pigs more resistant to pathogens and thus reducing antimicrobial use. Considering that prebiotic and antibacterial activities have also been observed for red and green seaweed polysaccharides, further studies are needed to evaluate their effects on pig health.

## Figures and Tables

**Table 1:** Polysaccharides and monosaccharides constituent of brown, green, and red seaweeds.

Chemical Constituent	Brown Seaweed	Green Seaweed	Red Seaweed
Polysaccharides	alginate, laminarin, fucoidan (sulphated), cellulose, mannitol	ulvan (sulphated), mannan, galactans (sulphated), xylans, starch, cellulose, lignin	carrageenans (sulphated), agar (sulphated), glucans (floridean starch), cellulose, lignin, funoran
Monosaccharides	glucose, galactose, fucose, xylose, uronic acid, mannuronic acid, guluronic acid, glucuronic acid	glucose, mannose, rhamnose, xylose, uronic acid, glucuronic acid	glucose, galactose, agarose
References	[12,13]	[12,13]	[12,13]

**Table 3:** Prebiotic effects of brown seaweed in sow.

Seaweed Supplement	Dose	Animal	Prebiotic Effect	Effect vs. Control, %	Reference
*Laminaria* spp. ^x^	LAM + FUC (1 + 0.8 g/day)	Sow lactation	Piglets (9 days after weaning): Villous height ileum Crypt depth duodenum Colon: *Bifidobacteria* spp. *Lactobacilli* spp. *E. coli*	+8% −5% −4% −2% (*p* < 0.10) −16% (*p* < 0.10)	[66]
*Laminaria* spp. ^x^	LAM + FUC (1 + 0.8 g/day)	Sow 107 d gestation and lactation	Piglets Colon: *E. coli* *Lactobacillus* spp. SCFA	−21% NS NS	[67]
*Laminaria* spp. ^x^	LAM + FUC (1 + 0.8 g/day)	Sow 83 d gestation and lactation	Sow: *Enterobacteriaceae* Piglets: *Enterobacteriaceae*	−9.2% NS	[68]
*Laminaria* spp. ^x^	LAM (1 g/day) FUC (0.8 g/day) LAM + FUC (1 + 0.8 g/day)	Sow 109 d gestation and lactation	Sow: *Lactobacillus* spp. *Enterobacteriaceae* Piglets: *Lactobacillus* spp.	−9% FUC +4.7% LAM + FUC +5.8% LAM	[69]
*Laminaria* spp. ^x^	Sow LAM + FUC (1 and 0.8 g/day) Piglets LAM + FUC (0.30 + 0.24 g kg^−1^)	Sow 107 d gestation and lactation Weaned piglets, 5.9 kg LW	Weaned piglets: heat-labile enterotoxin (caecum) enteroaggregative heat- stable enterotoxin (colon)	−14% −10%	[70]
*Laminaria* spp. ^x^	Sows LAM (1 g/day) Piglets LAM (0.3 g kg^−1^)	Sow 109 d g Gestation and lactation (LT) Weaned piglets, 5.6 kg LW (PW) Challenge *Salmonella Typhimurium* 10 d post weaning	Sow: *Lactobacillus* colon rectum LT + PW *E. coli* in rectum LT stimulated VFA production LT × PW SCFA and butyric acid	+4% +7% −13% +29% +24% +8.1%	[71]

FUC: fucoidan; LAM: laminarin; LT: lactation diet; LW: live weight; PW, post weaning diet; SCFA: short-chain fatty acid. ^x^ Extract from *Laminaria* spp. from BioAtlantis Ltd. (Clash Industrial Estate, Tralee, Co. Kerry, Ireland).

**Table 4:** Prebiotic effects of seaweed in weaned piglets.

Seaweed Supplement	Dose	Animal	Prebiotic Effect	Effect vs. Control, %	Reference
*Ascophyllum nodosum* ^a^	Dried intact (10–20 g kg^−1^)	Weaned piglets, 9 kg LW	10 g kg^−1^ *E. coli*: stomach small intestine	−42% −27%	[55]
*Ascophyllum nodosum* ^a^	Dried seaweed (5–10 g kg^−1^)	Weaned piglets, 6.59 kg LW	Proximal, distal small intestine and caecum	NS	[85]
*Ascophyllum nodosum*	Fucoidan rich extract (0.250 mg kg^−1^ FUC) ^n^	Weaned piglets, 8.5 kg LW	Small Intestinal Morphology Caecum: *Bacteroides* *Clostridia* Colon: SCFA	NS + + - +19%	[86]
*L. digitataL. hyperborea*	*L. digitata* extract (Ld) In diet LAM + FUC (0.170–0.136 g kg^−1^) *L. hyperborean* extract (Lh) In diet LAM + FUC (0.170–0.131 g kg^−1^) In diet Ld + Lh extracts (0.170–0.134 g kg^−1^)	Weaned piglets, 6.5 kg LW	Caecum *Lactobacillus* *Enterobatteriaceae* SCFA	−13% Lh −14% Ld −17% Lh + Ld −74% Ld −65% Lh + Ld +26% Ld	[87]
*Laminaria* spp.	Seaweed extract LAM + FUC ^c^ (0.300–0.236 kg^−1^)	Weaned piglets, 6.4 kg LW	Faecal *Lactobacilli* spp. *E. coli*	+2% (15% lactose) +5% (25% lactose) −8% (15% lactose) −29% (25% lactose)	[88]
*Laminaria* spp.	Seaweed extract LAM (0.300 g kg^−1^) ^y^ FUC (0.236 g kg^−1^) ^z^ LAM (0.3 g kg^−1^) + FUC (236 g kg^−1^)	Weaned piglets, 24 d age	Faecal *Lactobacillus* spp. *E. coli*	+5% −8% LAM	[89]
*Laminaria* spp.	Seaweed extract LAM (0.3 g kg^−1^) FUC (0.36 g kg^−1^) LAM + FUC (0.3 + 0.36 g kg^−1^)	Weaned piglets, 6.4 kg	Faecal *E. coli*	−8% LAM −12% LAM + FUC	[90]
*Laminaria* spp.	Seaweed extract LAM (0.30 g kg^−1^) ^y^ FUC (0.24 g kg^−1^) ^z^	Weaned piglets, 7.9 kg LW Challenge with *Salmonella typhimurium*	Caecum *Lactobacillus* spp. Acetic acid Propionic acid Butyric acid Colon: *Lactobacillus* spp. Acetic acid Propionic acid *Butyric acid*	+208% FUC +11% LAM FUC −15% LAM −22% FUC +23% FUC NS +10% FUC −24% FUC +31% FUC	[91]
*Laminaria* spp.	Seaweed extract LAM (0.30 g kg^−1^) FUC (0.24 g kg^−1^) LAM + FUC (0.30 + 0.24 g kg^−1^)	Weaned piglets, 6.8 kg LW	Colon prox: *Enterobacteriaceae* *Escherichia coli* Caecum SCFA	−15% FUC −15% LAM +15% LAM +8% FUC	[92]
*Laminaria* spp.	Seaweed extract LAM (0.15–0.30 g kg^−1^) FUC (0.24 g kg^−1^) LAM + FUC (0.15 + 0.24 and 0.30 + 0.24 g kg^−1^)	Weaned piglets, 6.8 kg LW	Faecal *Lactobacillus* spp. *Bifidobacterium* spp. *E. coli*	+8.6% FUC NS NS	[93]
*Laminaria* spp.	Seaweed extract LAM (0.30 g kg^−1^)	Weaned piglets, 6.9 kg LW	Faecal *E. coli*, *Lactobacillus* spp., *Bifidobacterium* SCFA	NS NS NS NS	[94]
*Laminaria* spp.	Seaweed extract In diet LAM + FUC (0.30 + 0.24 g kg^−1^) ^k^	Weaned piglets, 6.5 kg LW	Faecal *Lactobacillus* spp. *E. coli*	−11% NS	[95]
*Laminaria* spp.	21% CP + LAM 0.30 g kg^−1^ 18% CP + LAM 0.30 g kg^−1^	Weaned piglets, 6.5 kg	Colon *Enterobacteriaceae* *Lattobacillaceae*	NS NS	[96]
*Laminaria* spp.	Seaweed extract (0.30 g kg^−1^) ^r^	Weaned piglets, 8.4 kg LW	Caecum *Enterobatteciaceae* *Prevotella* Colon SCFA Acetic acid Butirric acid	- + + +21% +12% +53%	[97]
Brown seaweed	Alginic acid olisaccharides (ALGO) ^m^	Weaned piglets, 7.8 kg LW	Ileum *Bifidobacterium* *Lactobacillus* *E. coli* Caecum *Bifidobacterium* *Lactobacillus* *E. coli* Colon *Bifidobacterium* *Lactobacillus* *E. coli*	+13% +20% NS NS NS −13% NS NS −13%	[98]
Brown seaweed	Alginates oligosaccharides from brown seaweed (100 mg kg^−1^)	Weaned piglets, 6.2 kg LW	Caecum *Acetic acid* *Propionic acid* *Butyric acid* Colon *Acetic acid* *Propionic acid* *Butyric acid*	+13% +66% +12% NS NS +20%	[99]
*Ecklonia cava*	Seaweed (0.5–11.5 g kg^−1^) ^s^ FUC = 0.056–0.112–0.168 g kg^−1^	Weaned piglets, 7.8 kg LW	*Lactobacillus* spp. *Enterobacterium* spp.	+3% −4%	[100]

LAM: laminarin; FUC: fucoidan; SCFA: short-chain fatty acids. ^a^ Sum of non-starch polysaccharidemonomers and lignin = 503 g kg^−1^. ^c^ *Laminaria* extract with a content of 112 g kg^−1^ Laminarin and 89 g kg^−1^ Fucoidan. ^y^ *Laminaria* extract with a content of 990 g kg^−1^ Laminarin. ^z^ *Laminaria* extract with a content of 720 g kg^−1^ Fucoidan. ^k^ *Laminaria* extract with a content of 455 g kg^−1^ Laminarin and 360 g kg^−1^ Fucoidan. ^s^ *Ecklonia cava* with a content of 112 g kg^−1^ Fucoidan. ^r^ 653.2 g kg^−1^ Laminarin, 190 g kg^−1^ Fucoidan. ^m^ Alginic acid oligosaccharide (ALGO) is the lyase–lysate of alginic acid, which is a naturally occurring anionic polysaccharide isolated from the cell walls of seaweed polysaccharide isolated from the cell walls of seaweed. ^n^ 441 g kg^−1^ fucoidan, 25.9 g kg^−1^ laminarin, 135 g kg^−1^ alginates, 43.8 g kg^−1^ mannitol, 34.8 g kg^−1^ phlorotannins, 319.5 g kg^−1^ ash.

## Data Availability

Not applicable.
